# Hollow Zn_x_Cd_1−x_S nanospheres with enhanced photocatalytic activity under visible light

**DOI:** 10.1038/srep29997

**Published:** 2016-07-22

**Authors:** Ying Jin, Haoyun Zhang, Chuang Song, Lanfang Wang, Qingyi Lu, Feng Gao

**Affiliations:** 1 State Key Laboratory of Coordination Chemistry, Coordination Chemistry Institute, Collaborative Innovation Center of Advanced Microstructures, Nanjing National Laboratory of Microstructures, School of Chemistry and Chemical Engineering, Nanjing University, Nanjing 210093, P. R. China; 2 Department of Materials Science and Engineering, Collaborative Innovation Center of Advanced Microstructures, Nanjing National Laboratory of Microstructures, Nanjing University, Nanjing 210093, P. R. China; 3 College of Biological and Chemical Engineering, Anhui Polytechnic University, Wuhu 241000, Anhui P. R. China

## Abstract

Formation of solid solutions is a good strategy to acquire materials with special properties and bring forth new type of applications or enhance the performance of currently existing devices. In this study, hollow Zn_x_Cd_1−x_S nanospheres with different molar ratios were synthesized via a facile hydrothermal process. The products were fully characterized by X-ray diffraction, scanning electron microscopy, transmission electron microscopy, energy-dispersive X-ray spectroscopy, and UV-vis absorption spectroscopy. It was found that the photocatalysis performance of the as-prepared samples could be enhanced by formation of Zn_x_Cd_1−x_S solid solutions. In addition, their photocatalytic activities are dependent on the Zn/Cd molar ratios and nanostructures of Zn_x_Cd_1−x_S solid solutions. Hollow Zn_0.2_Cd_0.8_S spheres exhibit extremely high photocatalytic activity and good re-usability, and the photocatalytic conversion of RhB reaches as high as 96% after 50 min of irradiation.

Over the past decades, growing attention regarding environmental and energy problems have stimulated wide researches on solar energy untilization. Among them, using semiconductor photocatalysts to degrade organic dyes are extensively explored^1^,^2^. But most of semiconductor photocatalysts, such as TiO_2_ and ZnO, can only absorb UV light for photocatalytic activation due to their wide band gaps^3^,^4^. Since UV light accounts for a small portion (5%) of the solar spectrum as compared to visible light (52%)^5^, how to design high-performanced photocatalysts with suitable band gap for maximally utilizing visible light becomes important^6^.

II-VI semiconductor CdS with an appropriate band gap (2.4 eV) is regarded as an outstanding visible-light responsive material for photocatalysts^7^,^8^. However, the low separation efficiency of photogenerated electrons (e^−^) and holes (h^+^) and the fact that it is easily corroded^9^,^10^, limit its applications in solar conversion and environmental remediation. Enormous attention has been devoted to finding ways to overcome this weakness of CdS, such as coupling CdS with another semiconductor^11^,^12^, embedding CdS particles in graphene or polymer matrix^13^,^14^, and formation of solid solutions^15^,^16^. Among these strategies, formation of solid solutions is considered to be a good strategy to acquire materials with many unique properties^17^,^18^. Zn_x_Cd_1−x_S, a kind of ternary transition metal sulfides has fine and tunable absorption in the visible region of solar energy and excellent electrical conductibility, which has been recognized as compatible candidates for photocatalysts^19^,^20^. Various kinds of nanostructures of Zn_x_Cd_1−x_S, such as quantum dots^21^,^22^, nanoparticles^23^, nanowires^24^, nanobelts^25^, nanorods^26^, and nanoribbons^27^ have been prepared, while the hollow structured Zn_x_Cd_1−x_S have not been reported so far. Hollow structured materials show many advantages including porous structure, large surface area, high light-harvesting efficiency and fast mobility of charge carriers, and have promising application in the field of photocatalytic processes. Therefore, the design and fabrication of Zn_x_Cd_1−x_S hollow structure is one admirable strategy in photocatalysis.

Herein, we propose a simple straightfoward route to prepare Zn_x_Cd_1−x_S hollow spheres with the assistance of carboxymethyl cellulose (CMC) under hydrothermal conditions. This one-step route provides both the expected hollow structure and ternary chalcorenide Zn_x_Cd_1−x_S semiconductors with different molar ratios. More importantly, the as-obtained hollow Zn_x_Cd_1−x_S spheres exhibit great enhancement of photocatalytic activities for the degradation of Rhodamine B (RhB) under visible-light irradiation.

## Results and Discussion

**Figure 1:**
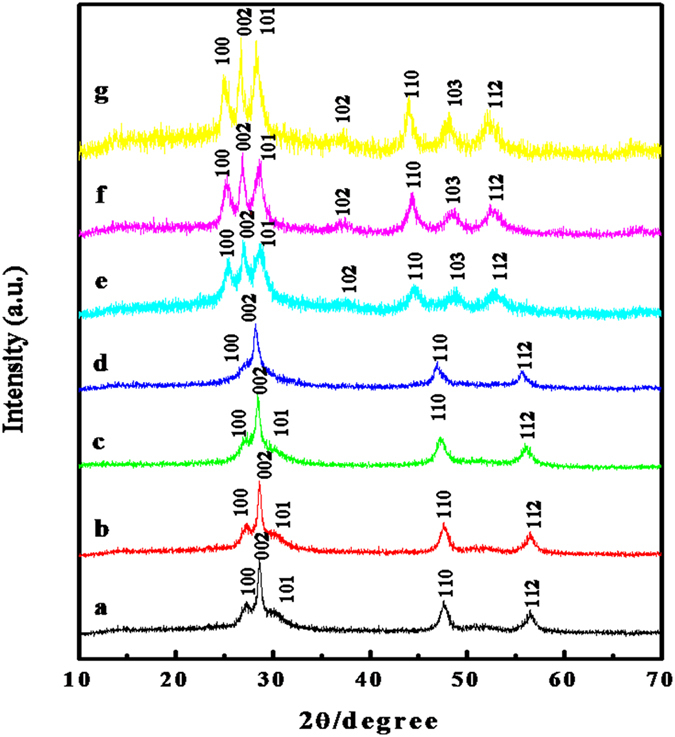
XRD patterns of Zn_x_Cd_1−x_S(0 ≤ x ≤ 1) samples (**a**) ZnS; (**b**) Zn_0.9_Cd_0.1_S; (**c**) Zn_0.8_Cd_0.2_S; (**d**) Zn_0.5_Cd_0.5_S; (**e**) Zn_0.2_Cd_0.8_S; (**f**) Zn_0.1_Cd_0.9_S; (**g**) CdS nanospheres.

A series of Zn_x_Cd_1−x_S hollow spheres were prepared with the assistance of carboxymethyl cellulose (CMC) and ammonia via a hydrothermal process, using zinc acetate, cadmium nitrate and l-cysteine as reactants. The molar ratios of Zn and Cd used were 10:0, 9:1, 8:2, 1:1, 2:8, 1:9 and 0:10, and the corresponding resulting products were labeled as ZnS, Zn_0.9_Cd_0.1_S, Zn_0.8_Cd_0.2_S, Zn_0.5_Cd_0.5_S, Zn_0.2_Cd_0.8_S, Zn_0.1_Cd_0.9_ S, and CdS, respectively. Figure 1 shows X-ray diffraction (XRD) patterns of the as-prepared ZnS, Zn_x_Cd_1−x_S and CdS samples. The diffraction peaks observed at 27.0°, 28.5° and 30.6° in Fig. 1a can be indexed to (100), (002) and (101) of the hexagonal structure of ZnS (JCPDS, 01-0677), while the diffraction peaks at 24.8°, 26.4° and 28.1° in Fig. 1g can be indexed to (100), (002) and (101) of the hexagonal structure of CdS (JCPDS, 41-1049), respectively. In the case of Zn_0.9_Cd_0.1_S, Zn_0.8_Cd_0.2_S, Zn_0.5_Cd_0.5_S, Zn_0.2_Cd_0.8_S, Zn_0.1_Cd_0.9_S products, from the XRD patterns in Fig. 1b–f, all the samples have a hexagonal crystal phase and all of the diffraction patterns are similar but with the obvious diffraction peak shifts. In a general way, since the ionic radius of Zn^2+^ is smaller than that of Cd^2+^, the increasing of Zn/Cd molar ratios leads to the shrinkage of the unit-cell volume of the Zn_x_Cd_1−x_S samples. Therefore, all the diffraction peaks shift to the higher angles with increasing the Zn^2+^ concentration as shown in Fig. 1, which indicates that the Zn_x_Cd_1−x_S solid solutions have been successfully prepared.

**Figure 2:**
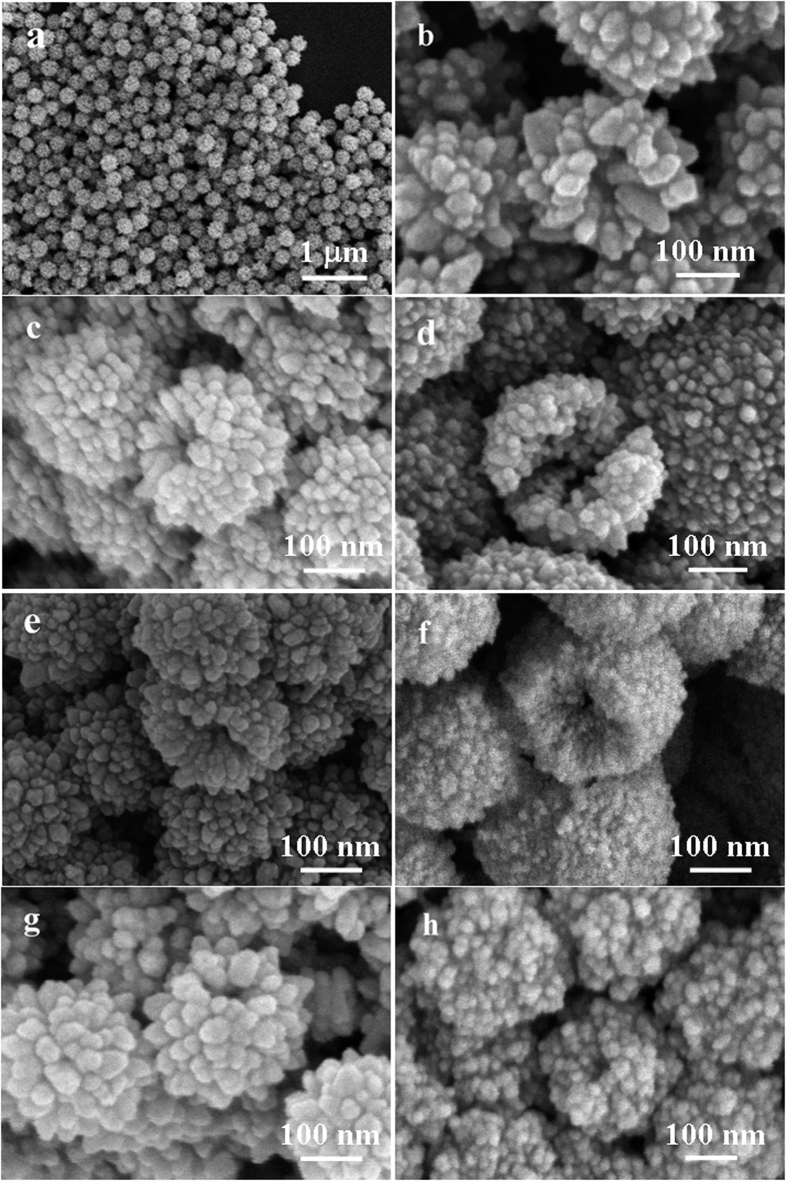
SEM images of (**a,b**) CdS; (**c**) Zn_0.1_Cd_0.9_S; (**d**) Zn_0.2_Cd_0.8_S; (**e**) Zn_0.5_Cd_0.5_S; (**f**) Zn_0.8_Cd_0.2_S; (**g**) Zn_0.9_Cd_0.1_S; (**h**) ZnS nanospheres.

The morphology of these Zn_x_Cd_1−x_S samples was investigated by scanning electron microscopy (SEM). Figure 2a shows a typical SEM image of the obtained CdS sample, which clearly displays that the sample consists of uniform nanospheres with an average diameter of 300 nm. Some open pores in the image obviously suggest the hollow nature of the nanospheres. A magnified SEM image (Fig. 2b) shows that the shells of these hollow nanospheres are rough and consist of many small CdS nanoparticles. Figure 2c~h show the SEM images of Zn_0.9_Cd_0.1_S, Zn_0.8_Cd_0.2_S, Zn_0.5_Cd_0.5_S, Zn_0.2_Cd_0.8_S, Zn_0.1_Cd_0.9_S and ZnS samples. It can be clearly observed that similar to CdS sample, with the increase of Zn content in the solid solutions, the obtained samples are all hollow structures with diameters of about 300 nm and the shell of the hollow structures are composed of small nanoparticles. In the process of reaction, both ammonia and carboxymethyl cellulose (CMC) play key roles in the formation of the Zn_x_Cd_1−x_S hollow structures. It is known that CMC is a kind of water soluble polyanionic compounds with a large number of carboxyl and hydroxyl groups. With the addition of CMC in the reaction system, the solution could be divided into numerous homogeneous “chambers”^28^, which can serve as soft templates to confine the growth of Zn_x_Cd_1−x_S during the growth process. Without the addition of CMC, only particles and irregular microspheres were obtained (Figure S1a,b). With the addition of CMC but in the absence of ammonia, uniform spheres can be achieved but these spheres are hard (Figure S1c,d). Even with the both addition of CMC and ammonia, if shorten the reaction time to one hour, only solid sphere products can be obtained (Figure S2). The “chambers” confine the crystal growth to form Zn_x_Cd_1−x_S nanoparticles and the assembly function of CMC further assembles these nanoparticles into uniform spheres. Ammonia can reacts with Zn^2+^ and Cd^2+^ to form [Zn(NH_3_)_4_]^2+^ and [Cd(NH_3_)_4_]^2+^ complexes, respectively. Under the hydrothermal conditions, after Zn_x_Cd_1−x_S spheres form, the existence of ammonia makes Zn_x_Cd_1−x_S dissolve to form Zn[(NH_3_)_4_]^2+^ and [Cd(NH_3_)_4_]^2+^ complexes. So, in the reaction system dissolution–precipitation dynamic equilibrium is presented:


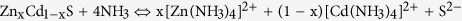


Under the dissolution-precipitation dynamic balance, based on the Ostwald’s ripening process, smaller Zn_x_Cd_1−x_S particles dissolve and the surface nanoparticles grow bigger. With continuous dissolve of the core Zn_x_Cd_1−x_S small particles, inner cavities eventually form, resulting in the formation of Zn_x_Cd_1−x_S hollow nanospheres.

**Figure 3:**
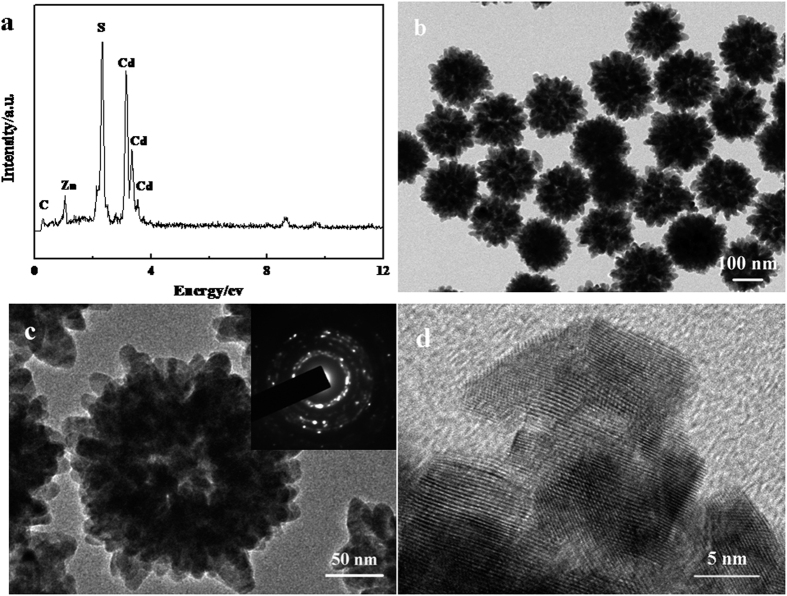
(**a**) EDS pattern; (**b,c**) TEM images and (**d**) HRTEM image of the as-prepared Zn_0.2_Cd_0.8_S hollow spheres. Inset in (c) SAED pattern of the Zn_0.2_Cd_0.8_S hollow sphere.

The composition, morphology and structure of the as-prepared Zn_x_Cd_1−x_S hollow spheres were further elucidated by energy dispersive X-ray spectroscopy (EDS) and transition electron microscopy (TEM). Figure 3 displays the EDS pattern, TEM and HRTEM images of the obtained Zn_0.2_Cd_0.8_S nanospheres. From the EDS pattern in Fig. 3a, the coexistence of Zn, Cd and S elements in the sample can be easily demonstrated, and quantitative analysis confirmed that the atom ratio of Zn/Cd/S is about 1:4:5, which is consistent with the used molar ratio of the reactants. The hollow structure was further confirmed by TEM image, in which a sharp contrast between the center and the boundary of the spheres can be clearly observed (Fig. 3b). Furthermore, a single hollow sphere TEM image (Fig. 3c) also reveals the hollow structure of Zn_0.2_Cd_0.8_S and the hollow spheres is composed of small nanoparticles. The selected area electron diffraction (SAED) pattern inset in Fig. 3c indicates the polycrystalline nature of the hollow Zn_0.2_Cd_0.8_S spheres. Figure 3d displays a high-resolution TEM (HRTEM) image of Zn_0.2_Cd_0.8_S sample, from which the lattice fringes are clearly visible and the spacing is about 0.338 nm, which corresponds to (002) lattice plane of hexagonal Zn_0.2_Cd_0.8_S.

**Figure 4:**
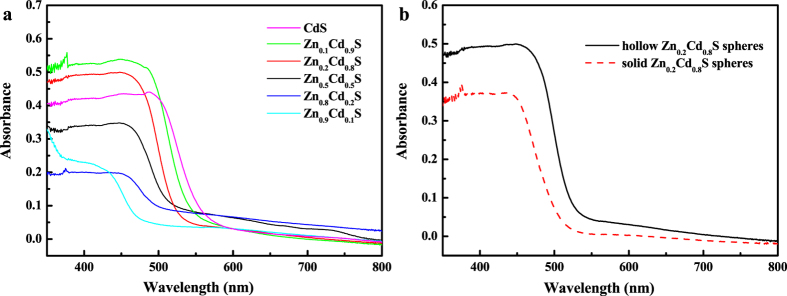
(**a**) UV-vis DRS of CdS and Zn_x_Cd_1−x_S (0 ≤ x < 1) samples; (**b**) UV-vis DRS of hollow Zn_0.2_Cd_0.8_S spheres and solid Zn_0.2_Cd_0.8_S spheres.

The optical property measurement of Zn_x_Cd_1−x_S hollow spheres and solid Zn_0.2_Cd_0.8_S sphere, using the UV−vis diffuse reflectance spectra (DRS), were shown in Fig. 4. DRS of Zn_x_Cd_1−x_S hollow spheres are shown in Fig. 4a. CdS hollow spheres shows a sharp absorption edge at around 560 nm, and the absorption edge for the Zn_x_Cd_1−x_S solid solutions with the increase of x value shows a blue-shift in turn relative to those of the hollow CdS spheres in the visible-light region, which further confirms the formation of the solid solution and indicates that the hollow Zn_x_Cd_1−x_S spheres have a bigger band gap than the CdS hollow spheres. Moreover, there is an enhanced light absorbance in the visible light region for Zn_0.2_Cd_0.8_S and Zn_0.1_Cd_0.9_S hollow spheres than CdS sample, which means a small x value would be beneficial to improve the absorption of visible light for Zn_x_Cd_1−x_S solid solutions. Plots obtained through the Kubelka-Munk transformation from the UV−vis diffuse reflectance spectra of all the prepared nanostructures are shown in Figure S3, from which the roughly estimated band gap energy (Eg) values are 2.29, 2.36, 2.42, 2.44, 2.48 and 2.66 eV for CdS, Zn_0.1_Cd_0.9_S, Zn_0.2_Cd_0.8_S, Zn_0.5_Cd_0.5_S, Zn_0.8_Cd_0.2_S and Zn_0.9_Cd_0.1_S, respectively. In Fig. 4b, hollow and solid Zn_0.2_Cd_0.8_S spheres’ DRS spectra were shown, which display that the absorption band edges are 534 and 520 nm for hollow and solid Zn_0.2_Cd_0.8_S sphere, respectively. The direct band-gap values of the hollow Zn_0.2_Cd_0.8_S spheres and solid Zn_0.2_Cd_0.8_S sphere can be estimated to be about 2.32 and 2.38 eV, respectively. Moreover, the visible light absorption of the hollow Zn_0.2_Cd_0.8_S spheres was clearly stronger than that of solid Zn_0.2_Cd_0.8_S spheres. The results show that both the Zn/Cd molar ratio and nanostructures of sample affect its optical properties and the formation of hollow solid solution would be beneficial to their photocatalytic performances.

**Figure 5:**
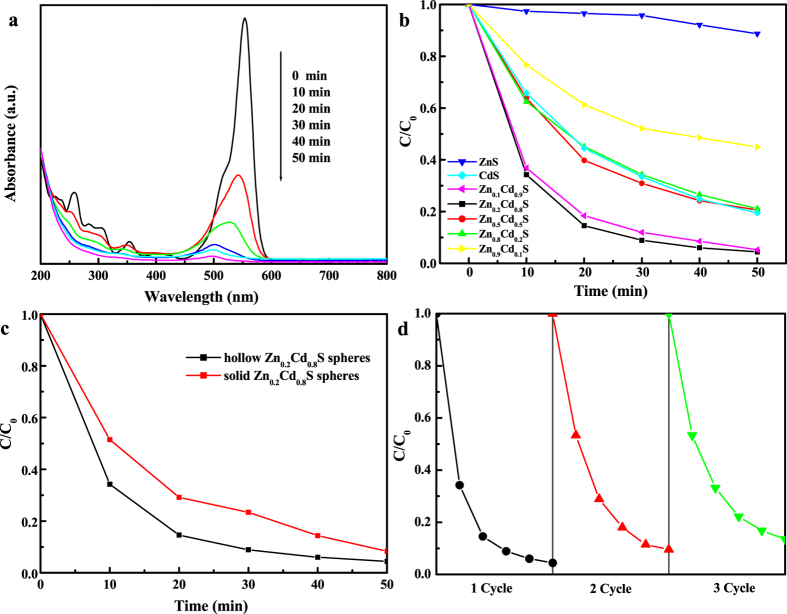
(**a**) Absorption spectra of dye solution after irradiation under visible-light for different time with the presence of Zn_0.2_Cd_0.8_S hollow spheres; (**b**) C/C_0_ versus time curves of dye solution under visible-light irradiation with different photocatalysts; (**c**) C/C_0_ versus time curves of dye solution under visible-light irradiation with hollow Zn_0.2_Cd_0.8_S spheres and solid Zn_0.2_Cd_0.8_S spheres samples as photocatalysts; (**d**) Three cycles using hollow Zn_0.2_Cd_0.8_S spheres photocatalyst under visible-light irradiation for 50 min.

In order to determine the potential applications of Zn_x_Cd_1−x_S hollow spheres, the photocatalytic performances of products were evaluated by the photodegradation of RhB, one of the common dyes, in aqueous solution under visible light. Figure 5a shows the absorption spectra of the dye solution in the presence of Zn_0.2_Cd_0.8_S hollow spheres under exposure to visible light. At the beginning, RhB shows a major absorption band at 554 nm. With the irradiation time prolonging, the absorption peak at 554 nm is blue-shift and decreases quickly. The shift of the absorption is caused by de-ethylation of RhB because of the attack by one of the active oxygen species on the N-ethyl group and RhB is transformed to rhodamine. Rhodamine can be then further decomposed due to the further destruction of the conjugated. During this process, the measured absorbance (A) would be the sum of the abosorbances of remaining RhB and its decomposition product. After 50 min degradation, the absorbance of the dye solution decreases to 4% of the original absorbance, which means both RhB and rhodamine have been almost decomposed and indicates Zn_0.2_Cd_0.8_S hollow spheres exhibit high photocatalytic activities for the decomposition of RhB.

Figure 5b presents the dye residue concentration ratio (C/C_0_, calculated from A/A_0_) with different samples as photocatalysts under different irradiation times. It can be clearly found that Zn_0.2_Cd_0.8_S and Zn_0.1_Cd_0.9_S hollow spheres show high photocatalytic activities compared to other samples, and the photocatalytic conversion of RhB with hollow Zn_0.2_Cd_0.8_S spheres reaches as high as 96% after 50 min of irradiation. The degradation of RhB over the as-prepared catalysts with a 50 minute irradiation time decreased in the order of Zn_0.2_Cd_0.8_S > Zn_0.1_Cd_0.9_S > Zn_0.5_Cd_0.5_S > Zn_0.8_Cd_0.2_S ≈ CdS > Zn_0.9_Cd_0.1_S > ZnS. Because of the large band gap of ZnS (3.7 eV), ZnS show relatively low photocatalytic activity under visible light. But what is worthy noting that most of hollow Zn_x_Cd_1−x_S spheres show enhanced activities than CdS hollow spheres, this result suggests that formation of Zn_x_Cd_1−x_S solid solutions can improve the photoactivity of CdS. The Zn/Cd molar ratio has a great effect on the photocatalytic activity of Zn_x_Cd_1−x_S solid solutions and Zn_0.2_Cd_0.8_S shows the best performances.

Not only Zn/Cd molar ratios of Zn_x_Cd_1−x_S samples affects the photocatalytic activities of the composite, but nanostructures of Zn_x_Cd_1−x_S also affect its photocatalytic performances. Figure 5c shows the curves of the dye residue concentration ratio as a function of irradiation time with different Zn_0.2_Cd_0.8_S nanostructures. The photocatalytic activity of hollow Zn_0.2_Cd_0.8_S sphere is clearly better than that of solid Zn_0.2_Cd_0.8_S spheres as shown in Fig. 5c. This could be ascribed to the unique hollow structure of Zn_0.2_Cd_0.8_S sample. The adsorption of contaminant molecules, light irradiation absorption, charge transportation and separation are three crucial factors for the photocatalytic activities of the composite^20^. Firstly, the hollow Zn_0.2_Cd_0.8_S sphere have much stronger visible light adsorbance and a smaller band gap according to UV-vis DRS (Fig. 4b). Secondly, the void interiors structure of Zn_0.2_Cd_0.8_S hollow spheres provides both inner and outer surfaces to interact with RhB molecules, allowing multiple reflections of visible light within the interior cavities and benefiting electrons and holes transportation and separation^21^. The BET measurements of the hollow and solid Zn_0.2_Cd_0.8_S spheres are provided in Figure S5. The BET surface area of the Zn_0.2_Cd_0.8_S hollow spheres is calculated to be 48 m^2^·g^−1^, a little larger than that of the Zn_0.2_Cd_0.8_S solid spheres (44 m^2^·g^−1^), further supporting the advantages of the hollow structure.

Stability and re-usability is an important standard to measure a photocatalyst. So, we further investigated the durability of hollow Zn_0.2_Cd_0.8_S sphere photocatalyst by collecting and reusing the photocatalyst for three cycles. Figure 5d shows the degradation percentage of dye solution maintained up to 90% even for the third cycle after 50 min photodegradation. The photocatalytic performance of the hollow Zn_0.2_Cd_0.8_S sphere has a small loss due to the loss of catalyst during each cycle of collecting and cleaning. These results indicate that Zn_0.2_Cd_0.8_S hollow sphere are photostable and reusable in the degradation of RhB.

## Conclusions

We demonstrated in this work an effective hydrothermal route for the synthesis of solid solution Zn_x_Cd_1−x_S hollow spheres. The prepared Zn_x_Cd_1−x_S hollow spheres show the enhanced photocatalytic activity for the degradation of RhB under visible light (λ > 420 nm) irradiation, and hollow Zn_0.2_Cd_0.8_S spheres were found to be highly efficient for RhB removal. Moreover, this hollow spherical Zn_x_Cd_1−x_S catalyst showed improved stability and would have great application potential in water treatment.

## Methods

### Synthesis of Zn_x_Cd_1−x_S hollow microspheres

In a typical synthesis, the appropriate molar ratios of Zn(CH_3_COO)_2_·2H_2_O and Cd(NO_3_)_2_·5H_2_O with the sum mole number of Zn^2+^and Cd^2+^ being 0.5 mmol were dissolved in a solution of sodium carboxymethyl cellulose (3 g/L in H_2_O, 18 mL) with magnetic stirring, then ammonia (25%, analytically pure, 3 mL) and l-cysteine (analytically pure, 3 mmol) were added under magnetic stirring. After stirring for 10 min, the mixture was transferred into a 40 mL autoclave. The autoclave was sealed, heated at 150 °C for 6 h, and cooled down to room temperature naturally. The precipitate was collected by centrifugation, washed with distilled water and ethanol several times, and then dried at 60 °C for 12 h. The same procedures were applied to synthesize CdS hollow spheres and ZnS sample, respectively.

### Characterizations

Powder X-ray diffraction (XRD) patterns were collected by using a Bruker D8 ADVANCE diffractometer with CuK_α_ radiation (λ = 1.5418 Ǻ). The morphology of the samples were characterized by scanning electron microscopy (SEM, Hitachi S-4800). Energy-dispersive X-ray spectroscopy (EDS) attached to the SEM instrument was used to analyze the composition of the Zn_x_Cd_1−x_S samples. Transmission electron microscopy (TEM), high-resolution TEM (HRTEM) images, and the corresponding selected area electron diffraction (SAED) were obtained with a JEOL JEM-2100 instrument transmission electron microscope at an acceleration voltage of 200 KV. UV/Vis diffusion reflectance spectra (DRS) of the samples were studied with a UV-3600 spectrophotometer (Shimadzu, Japan) and BaSO_4_ was used as the reference.

### Photocatalytic Studies

The photocatalytic activities of the synthesized samples for the photocatalytic decolorization of an aqueous solution of Rhodamine B (RhB) were evaluated as follows: the as-prepared sample (20 mg) was suspended in a aqueous solution of RhB (1.0 × 10^−5^ M, 80 mL) in a beaker at ambient temperature. A 300 W xenon lamp was used as the visible-light source with a cutoff filter to cut off the light below 420 nm. Before irradiation, the suspension was continuously magnetically stirred in the dark for 60 min to achieve the adsorption–desorption equilibrium between the photocatalyst powder and the dye. Then, the photocatalytic reaction was initiated. At specific time intervals, 4 ml of the aqueous solution was taken and separated through centrifugation (10000 rpm) for the absorbance measurements, which were recorded with a Hitachi U-3900 spectrophotometer.

## Additional Information

**How to cite this article**: Jin, Y. *et al*. Hollow Zn_x_Cd_1−x_S nanospheres with enhanced photocatalytic activity under visible light. *Sci. Rep.* **6**, 29997; doi: 10.1038/srep29997 (2016).

## Supplementary Material

Supplementary Information
