# Supplementary material for: Hollow ZnxCd1−xS nanospheres with enhanced photocatalytic activity under visible light
Source: Sci Rep. 2016 Jul 22;6:29997. doi: 10.1038/srep29997 (PMC4957231; doi:10.1038/srep29997)
Supplement: Supplementary Information [file srep29997-s1.doc]

**Supporting Information**

**Hollow Zn_x_Cd_1-x_S nanospheres with enhanced photocatalytic activity under visible light**

Ying Jin, Haoyun Zhang, Chuang Song, Lanfang Wang, Qingyi Lu,* Feng Gao*


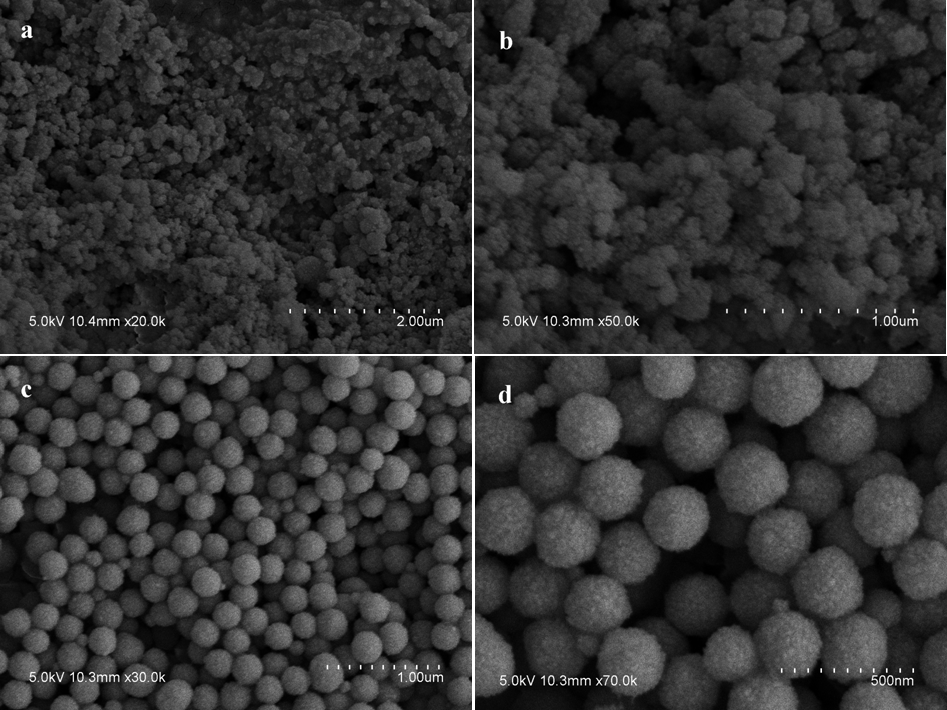


Figure S1 SEM images of the products prepared (a, b) without the addition of CMC and (c, d) without the addition of ammonia.

Figure S2 (a) XRD pattern; (b) EDS spectrum; (c) SEM image and (d) TEM image of the as-synthesized solid Zn_0.2_Cd_0.8_S spheres.

Figure S3 Plots obtained through the Kubelka-Munk transformation from the UV−vis diffuse reflectance spectra of all the prepared nanostructures

Figure S4 N_2_ adsorption/desorption isothermal curves of the obtained (a) hollow and (b) solid Zn_0.2_Cd_0.8_S spheres.
